# Comparing Enterovirus 71 with Coxsackievirus A16 by analyzing nucleotide sequences and antigenicity of recombinant proteins of VP1s and VP4s

**DOI:** 10.1186/1471-2180-11-246

**Published:** 2011-11-03

**Authors:** Yuyun Li, Runan Zhu, Yuan Qian, Jie Deng, Yu Sun, Liying Liu, Fang Wang, Linqing Zhao

**Affiliations:** 1 Graduate School, Peking Union Medical College, No. 9 Dongdansantiao Lane, Dongcheng District, Beijing, 100730, China; 2 Laboratory of Virology, Capital Institute of Paediatrics, No. 2 Yabao Road, Chaoyang District, Beijing, 100020, China

## Abstract

**Background:**

Enterovirus 71 (EV71) and Coxsackievirus A16 (CA16) are two major etiological agents of Hand, Foot and Mouth Disease (HFMD). EV71 is associated with severe cases but not CA16. The mechanisms contributed to the different pathogenesis of these two viruses are unknown. VP1 and VP4 are two major structural proteins of these viruses, and should be paid close attention to.

**Results:**

The sequences of *vp1s *from 14 EV71 and 14 CA16, and *vp4s *from 10 EV71 and 1 CA16 isolated in this study during 2007 to 2009 HFMD seasons were analyzed together with the corresponding sequences available in GenBank using DNAStar and MEGA 4.0. Phylogenetic analysis of complete *vp1s *or *vp4s *showed that EV71 isolated in Beijing belonged to C4 and CA16 belonged to lineage B2 (lineage C). VP1s and VP4s from 4 strains of viruses expressed in *E. coli BL21 *cells were used to detect IgM and IgG in human sera by Western Blot. The detection of IgM against VP1s of EV71 and CA16 showed consistent results with current infection, while none of the sera were positive against VP4s of EV71 and CA16. There was significant difference in the positive rates between EV71 VP1 and CA16 VP1 (χ^2 ^= 5.02, P < 0.05) as well as EV71 VP4 and CA16 VP4 (χ^2 ^= 15.30, P < 0.01) in the detection of IgG against recombinant proteins with same batch of serum samples. The sera-positive rate of IgG against VP1 was higher than that against VP4 for both EV71 (χ^2 ^= 26.47, P < 0.01) and CA16 (χ^2 ^= 16.78, P < 0.01), which might be because of different positions of VP1 and VP4 in the capsid of the viruses.

**Conclusions:**

EV71 and CA16 were highly diverse in the nucleotide sequences of *vp1s *and *vp4s*. The sera positive rates of VP1 and VP4 of EV71 were lower than those of CA16 respectively, which suggested a less exposure rate to EV71 than CA16 in Beijing population. Human serum antibodies detected by Western blot using VP1s and VP4s as antigen indicated that the immunological reaction to VP1 and VP4 of both EV71 and CA16 was different.

## Background

Hand, Foot and Mouth Disease (HFMD) is a mild exanthematous and febrile disease, which often poses a persistent global public health problem. In recent years, outbreaks of HFMD have been reported from many parts of the world such as Malaysia [1,2], Taiwan [3-6], Singapore [7], Mainland China [8], Brunei [9], Western Australia [10], the Unites States [11] and Germany [12]. The two major etiological agents for HFMD are Enterovirus 71 (EV71) and Coxsackievirus A16 (CA16), which belong to the *Enterovirus *genus of the *Picornaviridae *family [13] and usually co-circulate during HFMD outbreaks [4,14,15]. In addition to HFMD, EV71 is also associated with herpangina, myocarditis, encephalitis, aseptic meningitis, acute flaccid paralysis, and pulmonary oedema or haemorrhage. EV71 usually infects children, while sometimes it can infect adults by intra-familial transmission [16,17]. Generally, children and adults infected present different symptoms. Data from a recent study indicated that 21% of EV71-infected children experienced severe complications including central nervous system (CNS) complications and cardiopulmonary failure. By contrast, 53% of infected adults were asymptomatic, and all symptomatic adults recovered completely from uncomplicated illness [16]. However, there were several reports about adults infected with severe complications. It was reported that in November 2006, a 37-year-old woman suffered from acute encephalitis due to intra-familial transmission of EV71 [17]. In 2000 a 19-year-old man even died from EV71 encephalitis in Singapore [18]. CA16 appeared to have been attracting very little interest probably due to its association with often mild and benign clinical symptoms. Therefore, there had been much less data about CA16 than EV71.

Both EV71 and CA16 were divided into several subtypes by *vp1s *(referred to nucleotide sequences, the same below) or *vp4s *(referred to nucleotide sequences, the same below). Data from molecular epidemiological studies indicated that EV71 consisted of 3 genotypes A, B (B0~B5) and C (C1~C5) [14,19-24]. However, C4 was being proposed as genotype D recently [25,26]. Based on phylogenetic analysis of *vp4s*, CA16 was classified into three distinct genetic lineages A, B, and C. Lineage A was represented by only one isolate of the prototype G10 [27]. In a recent report, CA16 was divided into two distinct genogroups A and B based on *vp1s*, which was probably a more accurate description for *vp1s *containing more nucleotides and genetic information. The prototype G10 was the only member of genogroup A. Genogroup B was divided into two separate lineages (1 and 2) [28]. In fact, lineage B and C viruses in the analysis based on *vp4s *represented lineage B1 and lineage B2 viruses, respectively, in genogroup B as determined using complete *vp1 *sequences [28]. The capsids of both EV71 and CA16 consisted of 4 proteins: VP1 (referred to protein, the same below), VP2, VP3 and VP4 (referred to protein, the same below). VP1, VP2 and VP3 were on the outer part of the caspid while VP4 is on the inner part of it. It was believed that neutralization epitopes resided mainly on VP1, so most of researches had been focused on VP1, but only few on VP4.

Outbreaks of HFMD have occurred each year in Beijing recently [29] with various severity and outcomes of the disease which is associated with the predominant virus. The *vp1s *and *vp4s *of EV71 and CA16 isolated from the specimens collected from patients of HFMD in Beijing from 2007 to 2009 were sequenced and analyzed together with some corresponding sequences obtained from GenBank using DNAStar and MEGA 4.0 to analyze if the clinical manifestations of the children infected were related to the variation of the genes of the viruses. VP1 and VP4 encoding genes from field strains of EV71 and CA16 were cloned and expressed in *E. coli BL21 *cells. These expressed VP1s and VP4s were used as antigens to detect IgM and IgG antibodies in serum samples from children by Western Blot to analyze and compare their antigenicity and the prevalence of these two viruses.

## Results

### The epidemiologic characteristics of HFMD in children visiting our hospital from 2007 to 2009

From 2007 to 2009, no large epidemics of HFMD like some other provinces in China were reported in Beijing, but small local outbreaks with only a few cases with severe complications did occur. During these years, 535 clinical specimens were collected from 361 patients who visited the affiliated Children's Hospital to our institute, including 354 throat swabs and 181 vesicle fluids, and the case number each year was 59 (in 2007), 197 (in 2008) and 105 (in 2009). These specimens were subject to RT-PCR for EV71 and CA16 detection by using specific primers, followed by virus isolation with Vero cells. Out of these 535 clinical specimens, 336 (62.8%) virus strains were isolated. Co-infection by EV71 and CA16 was not found in these samples. Of the patients with molecularly confirmed EV71 or CA16 infection, the age ranged from 1 month to 15 years old, with 95% of the patients being less than 5 years old. The positive rates for EV71 in the cases from whom specimens were collected were 3.4% (2/59) in the year of 2007, 59.4% (117/197) in 2008 and 11.4% (12/105) in 2009. The positive rate for CA16 was 72.9% (43/59) in the year of 2007, 12.2% (24/197) in 2008 and 55.2% (58/105) in 2009. Therefore, the predominant etiological agent of HFMD in Beijing was CA16 in 2007 and 2009 but EV71 in 2008.

### Comparison of *vp1s *and *vp4s *among EV71 and CA16

The *vp1s *from 14 strains of EV71 isolated from clinical specimens in this study were sequenced and compared with *vp1s *from 21 strains of EV71 obtained from GenBank (see Additional file 1). Pairwise nucleotide and amino acid comparison of these sequences showed that the variability among them was small. The nucleotide identities among these sequences were 80.90%~99.70% and the deduced amino acid identities among them were 92.30%~100.00%, indicating that changes in amino acids were fewer than those in nucleotides. The *vp4s *from 10 out of these 14 field strains of EV71 were also sequenced and analyzed with *vp4s *from other 22 strains of EV71 obtained from GenBank (see Additional file 1). The nucleotide identities in these *vp4s *were similar to those in *vp1s *but the deduced amino acid sequences for these *vp4s *were 98.60%~100.00%. In addition, nucleotide sequence comparisons between sequences of EV71 isolated from mild cases and those of EV71 isolated from severe cases in the present study showed that there were no consistent divergences of nucleotides in *vp1s *or *vp4s *(data not shown). The *vp1s *from 14 strains of CA16 isolated from clinical specimens in this study were sequenced and analyzed with *vp1s *from 14 strains of CA16 obtained from GenBank (see Additional file 1). The nucleotide identities among them were 75.40% ~99.90% while the deduced amino acid identities of them were 91.20%~100.00%. The nucleotide identities among those CA16 VP4s were 80.20%~100.00% and the deduced amino acids of them were identical (Table 1).

**Table 1:** The nucleotide identities and amino acid identities for the corresponding genes

Sequence name	Number of strains	Nucleotide identity (%)	Amino acid identity (%)
EV71 vp1s	35	80.90~99.70	92.30~100.00
CA16 vp1s	28	75.40~99.90	91.20~100.00
EV71 vp1s/CA16 vp1s	35/28	62.00~66.80	70.00~72.70
EV71 vp4s	32	79.20~100.00	98.60~100.00
CA16 vp4s	15	80.20~100.00	100.00
EV71 vp4s/CA16 vp4s	32/15	64.30~73.90	78.30~79.70

The nucleotide sequences of *vp1s *between EV71 and CA16 were also compared using MegAlign of DNAStar. Both *vp1 *encoding gene from EV71 and CA16 was 891 nucleotides in length and the deduced amino acids of them were 297 in length. The identities of nucleotides for them were 62.50%~66.80% and the deduced amino acid identities for them were 70.00%~72.70%. The comparison between *vp4s *from EV71 and CA16 using MegAlign of DNAStar showed that the number of nucleotides was 207 in length and the deduced amino acids of them were 69 in length. The identities of nucleotides among them were 64.30%~73.90% and the identities of the deduced amino acids were 78.30%~79.70% (Table 1).

### Phylogenetic analysis of complete *vp1s *and *vp4s *from EV71 and CA16

**Figure 1:**
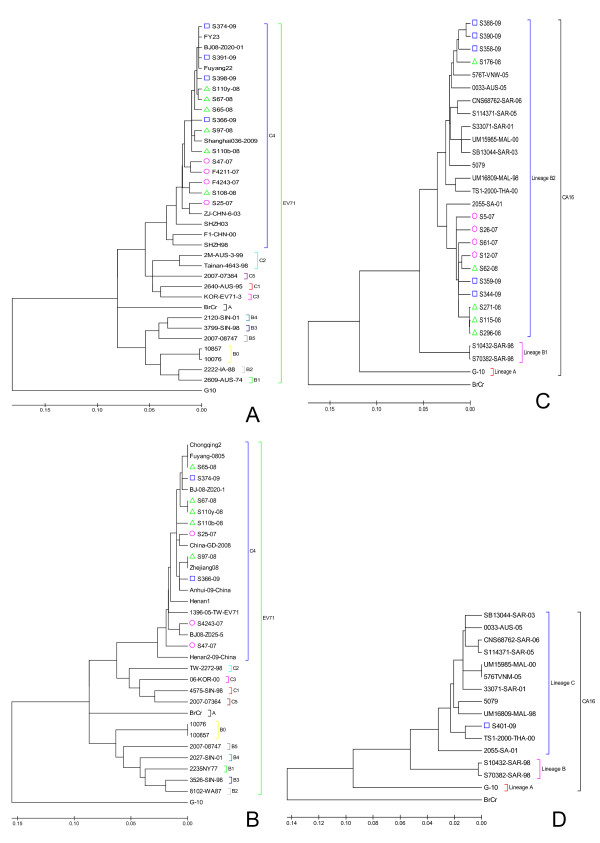
**Phylogenetic analysis based on EV71 *vp1s *(A), EV71 *vp4s *(B), CA16 *vp1s *(C) and CA16 *vp4s *(D)**. The unrooted phylogenetic trees were generated by the neighbor-joining method on the basis of a multiple alignment of the nucleotide sequences of EV71 *vp1s*, EV71 *vp4s*, CA16 *vp1s *and CA16 *vp4s*. The sequences in the dendrograms marked by red circle (○), green triangle (Δ) and blue square (□) were isolated in this research (additional file 2) while other sequences were obtained from GenBank (additional file 1). CA16 strain G-10 was used as an outgroup in Figure 1A and Figure 1B while EV71 strain BrCr was used as an outgroup in Figure 1C and Figure 1D.

Phylogenetic analysis of EV71 was based on the alignment of complete *vp1 *and *vp4 *gene sequences from EV71. A total of 36 strains were included in the phylogenetic analysis of the EV71 *vp1 *genes. Among them, *vp1s *from 14 EV71 field strains were sequenced in this study, 8 strains available in GenBank were reported in other studies in China, 13 strains obtained from GenBank were used as genotype reference and CA16 strain G-10 was used as an outgroup. Phylogenetic analysis of complete EV71 *vp1s *showed that these 14 EV71 strains isolated in this study from 2007 to 2009 was closest to C4 sub-genotype (Figure 1A). The phylogenetic analysis tree of EV71 *vp4s *(Figure 1B) including 10 strains isolated in this study, 9 strains reported in other studies in China and deposited in GenBank, 13 strains obtained from GenBank and used as genotype references and CA16 strain G-10 used as an outgroup, showed consistent result with that of phylogenetic analysis of complete *vp1s*. In the same way, *vp1s *from 14 CA16 strains isolated in this study, 14 sequences obtained from GenBank and EV71 strain BrCr used as an outgroup for phylogenetic tree analysis showed that lineage B2 of CA16 circulated in Beijing during 2007 to 2009 (Figure 1C). The phylogenetic analysis of complete CA16 *vp4s *including 1 sequences isolated in this study, 14 sequences obtained from GenBank and EV71 strain BrCr used as an outgroup showed that the CA16 viruses isolated in Beijing belonged to lineage C (Figure 1D), which was consistent with results from *vp1s*.

### Detection of IgM and IgG against EV71 and CA16 in serum samples by Western blot using expressed VP1 and VP4 as antigens

**Figure 2:**
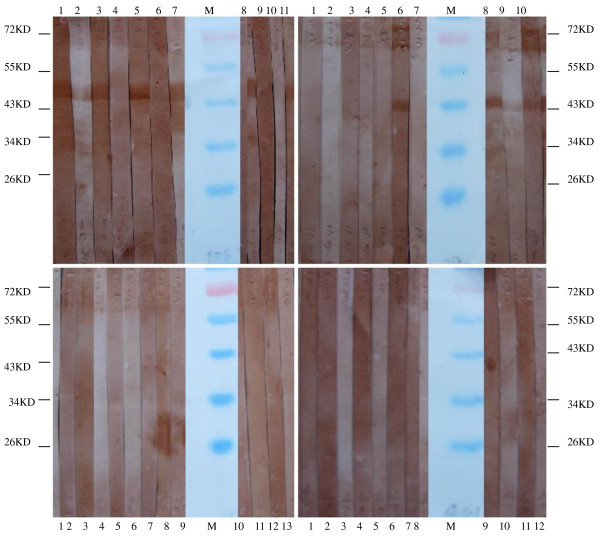
**Part of the results of the detection of IgM against s108 (EV71) VP1 (A), s67 (EV71) VP4 (B), s390 (CA16) VP1 (C) and s401 (CA16) VP4 (D) by Western Blot**. Western blot assay using goat anti-human IgM as secondary antibody. Lanes 1-7 in A, lanes 1-9 in B, lanes 1-7 in C and Lanes 1-8 in D represent immunoblotting with sera from patients with acute EV71 infection, and lanes 8-11 in A, lanes 10-13 in B, lanes 8-10 in C and Lanes 9-12 in D represent immunoblotting with sera from patients with acute CA16 infection. M represents molecular weight Marker(Fermentas, #SM0671).

The VP4s of EV71 (amplified from specimen s67) and CA16 (amplified from specimen s401) as well as VP1s of EV71 (amplified from specimen s108) and CA16 (amplified from specimen s390) were expressed in *E. coli BL21 *and used as antigen by Western Blot to detect specific IgM antibodies in serum samples collected from children with acute enterovirus (EV) infections (Figure 2). Out of 14 serum samples from children with acute EV71 infection, 12 were positive for VP1 of s108 (EV71) and 1 for VP1 of s390 (CA16). Out of 12 serum samples from children with acute CA16 infections, the number of positive serum samples for s108 VP1 and s390 VP1 were 3 and 7, respectively. This result suggested that VP1s from EV71 and CA16 could be used for the detection of IgM specific antibodies in serum samples from patients with acute infections (Table 2). When expressed VP4s of s67 (EV71) and s401 (CA16) were used as antigen to detect specific IgM, all of these 26 serum samples were negative, which raised the question about the antigenicity of the expressed VP4s from EV71 and CA16.

**Table 2:** Western Blot analysis of IgM in serum samples using s108 VP1 and s390 VP1 as antigens

proteins	Serum samples		sum
	positive	negative	
s108VP1	12	2	14
	*3*	*9*	*12*
s390VP1	1	13	14
	*7*	*5*	*12*

The data indicate the IgM detection in 14 sera from patients with acute EV71 infections by Western Blot using s108 VP1 and s390 VP1. The IgM detection in 12 sera from patients with acute CA16 infections by Western Blot using s108 VP1 and s390 VP1 is shown in italics.

**Figure 3:**
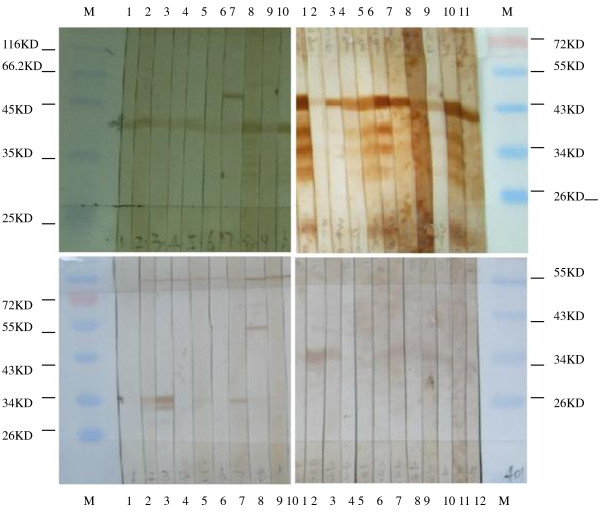
**Part of the results of the detection of IgG against s108 (EV71) VP1 (A), s67 (EV71) VP4 (B), s390 (CA16) VP1 (C) and s401 (CA16) VP4 (D) by Western Blot**. Western blot assay using goat anti-human IgG as secondary antibody. Lanes 1-10 in A, lanes 1-10 in B, lanes 1-11 in C and Lanes 1-12 in D represent immunoblotting with sera from adult for regular health check up. M represents molecular weight Marker (Fermentas, #SM0671).

These 4 expressed proteins were then used to detect specific IgG antibodies by Western Blot (Figure 3) in 189 serum samples, including 141 sera collected from adults for regular health check up and 48 sera from children without acute EV infections. The serum positive rate for IgG against EV71 VP1, CA16 VP1, EV71 VP4 and CA16 VP4 were 64.55% (122/189), 75.13% (142/189), 38.10% (72/189) and 58.20% (110/189), respectively. The data indicated that the expressed VP4s of EV71 and CA16 were of good antigenicity in the test of IgG specific antibodies. There was significant difference between the positive rates of IgG antibodies against VP1s of EV71 and CA16 (χ^2 ^= 5.02, P < 0.05), implying that these two proteins were not cross-reactive which was similar to the results from the study conducted by Shih et al [30]. The positive rates of IgG antibodies against VP4s of EV71 and CA16 (χ^2 ^= 15.30, P < 0.01) also suggested that there was no cross-reactivity between them. The sera-positive rate of EV71 VP1 was higher than that of EV71 VP4 (χ^2 ^= 26.47, P < 0.01) and in the same way the sera-positive rate of CA16 VP1 was higher than that of CA16 VP4 (χ^2 ^= 16.78, P < 0.01) (Table 3), which might be associated with the position of the proteins in the capsid of the virus, that was VP1 was located on the outside of the capsid while VP4 was located on the inside of the capsid. The serum IgG positive rates against VP1 and VP4 of EV71 were lower than those of CA16, suggesting that the exposure rate to EV71 was lower than that to CA16 in the population.

**Table 3:** Statistic analysis of the results of detection of IgG against 4 proteins by Western blot

	189 sera			
	Negative	Positive	**X**^**2**^	P
s108 VP1	67	122		
s390 VP1	47	142	5.02	P < 0.05
EV71 VP1	67	122		
EV71 VP4	117	72	26.47	P < 0.01
CA16 VP1	47	142		
CA16 VP4	79	110	16.78	P < 0.01
EV71 VP4	117	72		
CA16 VP4	79	110	15.30	P < 0.01

## Discussion

EV71 and CA16 were two of the members of the *Picornaviridae *family, whose genomes were characterized by a single positive-stranded genomic RNA. Due to their poor fidelity replication and frequent recombination, the genomes of EV71 and CA16 mutated at a high rate. Different genotypes and sub-genotypes of these 2 viruses had alternated and co-circulated in the Asia-Pacific region, leading to repeated outbreaks of HFMD. The first reported large, severe and devastating HFMD epidemic occurred in Taiwan region in 1998 including about 130000 cases of HFMD, among whom 405 patients were severe and 78 died [3,4,31]. In 2000, there was another report of outbreak, with 80677 cases of HFMD and 41 deaths there [6]. From February to August in 2006, Brunei with a population of about 370000 experienced its first reported major outbreak of EV71. More than 1681 children were affected, with 3 deaths resulting from severe neurologic complications [9]. In Mainland China, HFMD broke out repeatedly in recent years. There were 83344, 488955 and 1155525 cases in the nationwide in 2007, 2008 and 2009, respectively, reported by the Ministry of Health, the People's Republic of China. The corresponding deaths for these years were 17, 126 and 353, respectively. It suggested that HFMD had been becoming a more and more serious public health problem in China.

In Beijing, no large HFMD epidemic has occurred so far, but sporadic infections are common. In 2007 and 2009, the predominant etiological agents of HFMD in Beijing were CA16 while the main etiological agent was EV71 in 2008. In general, comparison for nucleotides among *vp1s *or *vp4s *of EV71 indicated that the nucleotide identity of these sequences from strains isolated in the same year was higher than that of those sequences from strains isolated in the different years, and the nucleotide identity of these sequences isolated in this study was higher than that of those sequences reported in other parts of Mainland China and especially other countries of the world. However, it was not necessarily true. For example, the nucleotide identity of s374 *vp4 *isolated in 2009 and those isolated in 2008 in this research was higher than that of s374 *vp4 *and s366 *vp4 *isolated in the same year of 2009. This suggested that the transmission of EV71 was not strictly regional and temporal restriction. In addition, the nucleotide comparison also indicated that the severity of patients' illness caused by EV71 infection seemed not to be correlated with the sequence mutations in *vp1 *or *vp4*. The phylogenetic data in this study indicated that C4 of EV71 and lineage B2 (C) of CA16 had been circulating in Beijing in these 3 years and major mutations were not observed in these virus strains, which was similar to the results reported by other parts of Mainland China [14]. In Mainland China there were no subgenotypes other than C4 of EV71 reported which seemed to be regionally and temporally restricted, but it was not true for the lineage of CA16. In Shenzhen, lineage B1 and B2 co-circulated in 1999 and 2000, but only lineage B2 was found from 2001 to 2004. In other parts of the world, the transmission of genotypes of EV71 and lineages of CA16 showed a different trend. For example, in Malaysia EV71 outbreaks occurred in 1997 and 2000, mainly associated with genotypes B3 and B4 alternating in the 2 years[32,22], and lineage B1 and B2 of CA16 coexisted in 2000 and 2003[33]. In Taiwan region, EV71 epidemics were associated with genotype C2 and B4.

The overall sero-positive rates of VP1 of EV71 and CA16 in this research were 64.55% and 75.13%, respectively, which were higher than those reported by Rabenau et al, whose data showed 42.8% for EV71 and 62.9% for CA16 for those individuals ≥ 1 years old [34]. The difference of sero-positive rate in these two studies might be caused by the variety of the detection method used or age group of the participants. Nevertheless, both results from our study and Rabenau' suggested that the exposure rate of CA16 was higher than that of EV71 in the population.

EV71 other than CA16 was the cause of severe cases of HFMD in young children. Generally the severity of the patients infected by viruses was associated with 2 factors: host and virulence of the virus [4]. When HFMD outbreaks were caused by EV71, there would be some severe cases and even deaths [3,6]. CA16 was often associated with mild and benign clinical symptoms. Then the pathogenicity of EV71 should be stronger than that of CA16. EV71 and CA16 shared a lot in some characteristics. For example, both of them belonged to *Enterovirus *A and had a genome of about 7.4 k bp in length. The caspids of them consisted of 4 proteins: VP1, VP2, VP3 and VP4. Both of them could cause HFMD. However, there were also many differences between them. In this study, we designed experiments to compare EV71 and CA16 in some aspects and tried to find some of the differences. The nucleotide identities of VP1 between them were less than 66.80% and the identities of deduced amino acids were no more than 72.70%. Although VP4s from them were much conserved, there were still some differences in nucleotides and the deduced amino acids. The nucleotide identities of VP4s between them were 64.30%~73.90% and the deduced amino acids identities were 78.30%~79.70%. There were also some differences in inducing IgG in host's sera against VP1 and VP4 between EV71 and CA16. The sera-positive rate of EV71 VP1 in the population was lower than that of CA16 VP1 and similarly the sera-positive of EV71 VP4 was lower than that of CA16 VP4, for which there might be 2 reasons. One was that the exposure rate of EV71 might be lower than that of CA16. Another was that it was more difficult to induce IgG against EV71 than CA16 in hosts' sera, which might be associated with the different symptoms caused by EV71 and CA16. When the specific IgM against VP4s of these two viruses were tested in serum specimens collected from children currently infected with these two viruses, none of these sera showed positive reaction.

## Conclusions

EV71 and CA16 were highly diverse in the nucleotide sequences of *vp1s *and *vp4s*. The severity of illness of EV71 infected was not associated with the sequence variation of *vp1s *or *vp4s*. The sera positive rates of VP1 and VP4 of EV71 were lower than that of CA16, suggesting less exposure rate to EV71 than CA16 in Beijing population. The detection of serum antibodies by Western blot using VP1s and VP4s as antigens indicated that the immunological reaction to VP1 and VP4 of both EV71 and CA16 was different. IgM against VP1 but not VP4 was generated in children after acute infections, which needs to be clarified further.

## Methods

### Clinical specimens and isolation of viruses

Throat swabs and vesicle fluids were collected from infants and children with clinical diagnosis of HFMD or suspected EV infection who visited the Affiliated Children's Hospital to Capital Institute of Paediatrics during the HFMD seasons of year 2007 to 2009. The specimens were inoculated in Vero cells after being delivered to the Laboratory of Virology, and CPE were observed by microscopy everyday. When the CPE reached ++++, the isolates were harvested and stored at-80°C until use.

### Serum specimens

Serum specimens for the detection of IgM antibodies against the expressed VP1s and VP4s were collected from infants and children with acute EV infection, including 14 from children with acute EV71 infection and 12 from children with acute CA16 infection identified by RT-PCR, virus isolation from throat swabs and vesicle fluids, and immnofluorescence staining of IgM against EV71 or CA16 in sera (data not shown). Another batch of 189 sera were collected for the detection of IgG antibodies against the expressed proteins, including 141 from adults for regular health check up and 48 children without acute EV infections.

The study was performed according to the Declaration of Helsinki II and approved by Ethics Committee of Capital Institute of Paediatrics and written informed consent was obtained from all patients or from their caretakers.

### Identification of EV71 and CA16 from clinical specimens and isolated viruses by RT-PCR

RNAs were extracted from clinical specimens and isolated virus strains using Trizol (Invitrogen, USA) following the instructions provided by manufacture. RT-PCR was carried out to identify EV71 and CA16 in the specimens and virus isolates. Viral cDNAs were generated using random primer (Invitrogen, USA) and M-MLV (Invitrogen, USA) by reverse transcription. EV consensus primers, EV71 and CA16 specific primers were synthesized according to Perara D's [33] and Singh S' [35], and used to detect EV71 and CA16 by PCR as described by our group previously [29]. The PCR products were analyzed by electrophoresis in a 2% agarose (GibcoBRL, US) gel and visualized by staining the gels with ethedium bromide.

### Cloning and sequencing of *vp1s *and *vp4s*

cDNA products from several virus isolates were selected (see Additional file 2) and amplified by PCR with primers designed using primer premier 5.0 (Table 4). The PCR cycling conditions for amplifying EV71 *vp1s*, EV71 *vp4s *and CA16 *vp4s *consisted of 4 min at 94°C, followed by 35 cycles of 94°C 30 s, 52°C 30 s, 72°C 1 min, and then 72°C for 7 min. The steps for amplifying EV71 *vp4s *were the same as those for amplifying the other 3 protein genes except for annealing temperatures at 55°C for 30 s. Agarose gel electrophoresis and EasyPure Quick Gel Extraction Kit (Trans Gen Biotech, China) were used to purify those amplified products. The purified products were ligated to pGEM-T cloning vector (Promega, USA) for transformation into competent DH5α cells. Positive clones were identified by White-Blue colony selection and sequencing (Invitrogen Co).

**Table 4:** Primers used for cloning and sequencing

primers	sequences	fragments (bp)
EV71-VP1-1F	5'-TGAAGTTRTGYAAGGATGC-3'	
EV71-VP1-1R	5'-CCACTCTAAAATTRCCCAC-3'	993
EV71-VP4-1F	5'-CTACTTTGGGTGTCCGTGTT-3'	
EV71-VP4-1R	5'-GGGAACTTCCAGTACCATCC-3'	655
CA16-VP1-1F	5'-ACTATGCAAGGACACWGAG -3'	
CA16-VP1-1R	5'- CAGTGGTGGAAGAGACTAAA-3'	1076
CA16-VP4-1F	5'- GGCTGCTTATGGTGACAA-3'	
CA16-VP4-1R	5'- CATGGGAGCTATGGTGAC-3'	1090

F referred as forward primer and R referred as reverse primer.

### Expression and Purification of VP1s and VP4s

The pET-30a vector with an N-terminal His·Tag/thrombin/S·Tag™/-enterokinase configuration plus an optional C-terminal His·Tag sequence with endonuclease sites of BamHⅠand XhoⅠand the pGEX-4T-1 vector with an N-terminal GST (glutathione S-transferase) ·Tag/thrombin configuration with endonuclease sites of EcoRⅠ and XhoⅠwere used for expressing VP1s and VP4s, respectively. The virus isolates selected for expression were s67 (for VP4 of EV71), s108 (for VP1 of EV71), s390 (for VP1 of CA16) and s401 (for VP4 of CA16). The genes were purified with agarose gel electrophoresis and EasyPure Quick Gel Extraction Kit after being amplified by PCR with corresponding primers (Table 5). The cycling condition for amplifying VP1s of EV71 and CA16 consists of 95°C for 4 min, followed by 35 cycles of 95°C 30 s, 55°C 30 s, 72°C 1 min, and then 72°C for 7 min. The steps for amplifying VP4 of EV71 and CA16 were the same as those for amplifying the VP1s, except that the annealing temperatures were 50°C and 57°C respectively.

**Table 5:** Primers designed for the expression of VP1s and VP4s

primers	sequences	endonuclease	fragments (bp)
EV71-VP1-2F	5'- ACGGATCCGGAGATAGGGTGGCAGAT -3'	BamHⅠ	
EV71-VP1-2R	5'- CCTCTCGAGTTAAAGAGTGGTGATCGCTGTG -3'	XhoⅠ	911
EV71-VP4-2F	5'-CCGAATTCATGGGCTCGCAAGTG-3'	EcoRⅠ	
EV71VP4-2R	5'-TCTCTCGAGTTACTTCAGCGGCGCTGC-3'	XhoⅠ	227
CA16-VP1-2F	5'- GAAGATCTGGGGGATCCTATCGCAGAC-3'	BamHⅠ	
CA16-VP1-2R	5'- CCGCTCGAGTTACAGTGTTGTTATCTTG-3'	XhoⅠ	912
CA16-VP4-2R	5' -CGCCGAATTCATGGGGTCACAAGT -3'	EcoRⅠ	
CA16-VP4-2F	5'- TATCTCGAGTTACTTGAGCGGCGGG -3'	XhoⅠ	229

F referred as forward primer and R referred as reverse primer.

These genes as well as plasmid DNAs of PET30a and pGEX-4T-1 were digested with corresponding endonuclease (Promega, table 2) and ligated, followed by transformation into competent *BL21 *cells. After being verified by sequencing, these recombinants were induced with 1 mM isopropyl β-thiogalactopyranoside (IPTG) for 3 hours. The cells were incubated on ice for 30 min and harvested by centrifugation at 5000 g and 4°Cfor 5 min. The pellets containing VP1s were re-suspended in Buffer A (50 mM Tris-HCL PH 8.0, 150 mM Nacl, 2 mM Cacl_2_, 0.1% Triton-X-100), lysed by sonication for 5 min and centrifuged at 11,300 g and 4°C for 15 min. The supernatants were removed and the pellets were washed with Buffer B (50 mM Tris-Hcl PH 8.0, 1 mM EDTA, 0.2% Triton-X-100, 4 M Urea) at 11,300 g and 4°C for 15 min for twice. The pellets were re-suspended in Wash Buffer (0.1 M NaH_2_PO_4_, 10 mM Tris-Hcl, 8 M Urea) and incubated on ice for 2 hours. The supernatants were clarified by centrifugation at 11,300 g and 4°C for 15 min and loaded on columns for purification. The pellets containing VP4s were re-suspended in PBS (140 mM Nacl, 2.7 mM Kcl, 10 mM Na_2_HPO_4_, and 1.8 mM KH_2_PO_4_) and sonicated for 5 min. The supernatants were separated from the pellets by centrifugation at 10000 g and 4°C for 10 min and harvested, and the pellets were re-suspended in PBS containing 8 M Urea and mixed with the supernatants harvested. The mixtures were clarified by centrifugation at 10000 g and 4°C for 10 min and the supernatants were loaded on columns for purification. VP1s were purified by nickel-nitrilotriacetic acid (Ni-NTA) agarose (Qiagen, Valencia, CA) and VP4s were purified by Glutathione Sepharose™ 4B (GE Healthcare, Sweden) following the instructions of manufactures, respectively.

### Detection of IgM/IgG against expressed VP1s and VP4s in serum samples by Western Blot

The purified proteins of VP1s and VP4s were separated by SDS-PAGE using 12% polyacrylamide gel and electro-transferred onto nitrocellulose membranes according to standard procedures (Bio-Rad Laboratories). The transferred membranes were blocked with 5% non-fat milk in PBS, sliced into strips, and probed by sera. The dilutions of sera were 1:10 and 1:200 for the detection of IgM and IgG respectively. The secondary antibodies were goat anti-human IgM conjugated with horseradish-peroxidase (Jackson ImmunoResearch Laboratories, Inc., USA) and goat anti-human IgG conjugated with horseradish-peroxidase (Jackson ImmunoResearch Laboratories, Inc., USA), respectively. The membranes were developed with 3, 3'-diaminobenzidine (DAB, AMRESCO Inc., USA) colour developing reagent.

### Data analysis

Sequence analyses were performed using DNAStar and MEGA 4.0. The MagAlign of DNAStar was used to compare nucleotides and deduced amino acids by sequence distances and manual calculation. A phylogenetic tree was constructed by the neighbour-joining method after estimation of genetic distance using the p-distance method [36]. A bootstrapping test was performed 1,000 pseudo replicate data sets. The data about the detection of IgG in sera by Western Blot were analyzed by chi-square method (χ2 test). P < 0.05 is the level for significant difference.

## Competing interests

The authors declare that they have no competing interests.

## Authors' contributions

YL carried out nucleotide sequencing, expression of VP4 proteins, Western blot, data analysis, and drafting the manuscript. RZ performed the design of the experiment, nucleotide sequencing, expression of VP1 proteins, Western blot, data analysis and revising of the manuscript. The corresponding author, YQ is the PI of the project, participated in study design and coordination and performed data analysis and revising the manuscript. JD, YS, LL, FW and LZ were involved in the collection of samples, virus isolation and RT-PCR for identification of the isolates. All authors have read and approved the final manuscript.

## Supplementary Material

Additional file 1 **The strains obtained from GenBank referred in this research**. Click here for file

Additional file 2 **Virus strains cloned and sequenced in this research**. Click here for file

## References

[B1] Abubakar S Chee HY Shafee N Chua KB Lam SK Molecular detection of enteroviruses from an outbreak of hand, foot and mouth disease in Malaysia in 1997 Scand J Infect Dis 1999 31 331 335 10.1080/00365549950163734 10528868

[B2] Shimizu H Utama A Yoshii K Yoshida H Yoneyama T Sinniah M Yusof MA Okuno Y Okabe N Shih SR Chen HY Wang GR Kao CL Chang KB Miyamura T Hagiwara A Enterovirus 71 from fatal and nonfatal cases of hand, foot and mouth disease epidemics in Malaysia, Japan and Taiwan in 1997-1998 Jpn J Infect Dis 1999 52 12 15 10808253

[B3] Ho M Chen ER Hsu KH Twu SJ Chen KT Tsai SF Wang JR Shih SR An epidemic of enterovirus 71 infection in Taiwan. Taiwan Enteroviurs Epidemic Working Group N Engl J Med 1999 341 929 935 10.1056/NEJM199909233411301 10498487

[B4] Lin TY Twu SJ Ho MS Chang LY Lee CY Enterovirus 71 outbreaks, Taiwan: occurrence and recognition Emerg Infect Dis 2003 9 291 293 12643822 10.3201/eid0903.020285 PMC2963902

[B5] Lu CY Lee CY Kao CL Shao WY Lee PI Twu SJ Yeh CC Lin SC Shih WY Wu SI Huang LM Incidence and case-fatality rates resulting from the 1998 enterovirus 71 outbreak in Taiwan J Med Virol 2002 67 217 223 10.1002/jmv.2210 11992582

[B6] Wang JR Tuan YC Tsai HP Yan JJ Liu CC Su IJ Change of major genotype of enterovirus 71 in outbreaks of hand-foot and-mouth disease in Taiwan between 1998 and 2000 J Clin Microbiol 2002 40 10 15 10.1128/JCM.40.1.10-15.2002 11773085 PMC120096

[B7] Ahmad K Hand, foot and mouth disease outbreak reported in Singapore Lancet 2000 356 1338 11073035 10.1016/S0140-6736(05)74253-7

[B8] Ding NZ Wang XM Sun SW Song Q Li SN He CQ Appearance of mosaic enterovirus 71 in the 2008 outbreak of China Virus Res 2009 145 1 157 161 10.1016/j.virusres.2009.06.006 19540282

[B9] AbuBakar S Sam IC Yusof J Lim MK Misbah S Enterovirus 71 outbreak, Brunei Emerg Infect Dis 2009 15 79 82 10.3201/eid1501.080264 19116058 PMC2660687

[B10] McMinn P Stratov I Nagarajan L Davis S Neurological manifestations of enterovirus 71 infection in children during an outbreak of hand, foot, and mouth disease in Western Australia Clin Infect Dis 2001 32 236 242 10.1086/318454 11170913

[B11] Alexander JP Jr Baden L Pallansch MA Anderson LJ Enterovirus 71 infections and neurologic disease-United States, 1977-1991 J Infect Dis 1994 169 905 908 10.1093/infdis/169.4.905 8133108

[B12] Kehle J Roth B Metzger C Pfitzner A Enders G Molecular characterization of an Enterovirus 71 causing neurological disease in Germany J Neurovirol 2003 9 126 128 12587076 10.1080/13550280390173364

[B13] Oberste MS Peñaranda S Maher K Pallansch MA Complete genome sequences of all members of the species Human enterovirus A J Gen Virol 2004 85 Pt 1597 1607 15166444 10.1099/vir.0.79789-0

[B14] Li Linlin He Yaqing Yang Hong Zhu Junpin Xu Xingye Dong Jie Zhu Yafang Jin Qi Genetic Characteristics of Human Enterovirus 71 and Coxsackievirus A16 Circulating from 1999 to 2004 in Shenzhen, People's Republic of China J Clin Microbiol 2005 43 8 3835 3839 10.1128/JCM.43.8.3835-3839.2005 16081920 PMC1233905

[B15] Podin Y Gias EL Ong F Leong YW Yee SF Yusof MA Perera D Teo B Wee TY Yao SC Yao SK Kiyu A Arif MT Cardosa MJ Sentinel surveillance for human enterovirus 71 in Sarawak, Malaysia: lessons from the first 7 years BMC Public Health 2006 6 180 10.1186/1471-2458-6-180 16827926 PMC1543637

[B16] Chang LY Tsao KC Hsia SH Shih SR Huang CG Chan WK Transmission and clinical features of enterovirus71 infections in household contacts in Taiwan JAWA 2004 291 222 227 10.1001/jama.291.2.222 14722149

[B17] Hamaguchi Tsuyoshi Fujisawa Hironori Sakai Kenji Okino Soichi Kurosaki Naoko Nishimura Yorihiro Shimizu Hiroyuki Yamada Masahito Acute encephalitis caused by intrafamilial transmission of enterovirus 71 in adult Emerg Infect Dis 2008 14 5 828 830 10.3201/eid1405.071121 18439374 PMC2600258

[B18] Chan KP Goh KT Chong CY Teo ES Lau G Ling AE Epidemic hand, foot, and mouth disease caused by human enterovirus 71, Singapore Emerg Infect Dis 2003 9 78 85 12533285 10.3201/eid1301.020112 PMC2873753

[B19] Van der Sanden S Koopmans M Uslu G van der Avoort H Dutch Working Group for Clinical Virology Epidemiology of enterovirus 71 in the Netherlands, 1963 to 2008 J Clin Microbiol 2009 47 9 2826 2833 10.1128/JCM.00507-09 19625480 PMC2738086

[B20] Brown BA Oberste MS Alexander JP Jr Kennett ML Pallansch MA Molecular epidemiology and evolution of enterovirus 71 strains isolated from 1970 to 1998 J Virol 1999 73 9969 9975 10559310 10.1128/jvi.73.12.9969-9975.1999 PMC113047

[B21] Brown BA Pallansch MA Complete nucleotide sequence of enterovirus 71 is distinct from poliovirus Virus Res 1995 39 195 205 10.1016/0168-1702(95)00087-9 8837884

[B22] McMinn P Lindsay K Perera D Chan HM Chan KP Cardosa MJ Phylogenetic analysis of enterovirus 71 strains isolated during linked epidemics in Malaysia, Singapore, and Western Australia J Virol 2001 75 7732 7738 10.1128/JVI.75.16.7732-7738.2001 11462047 PMC115010

[B23] Mizuta K Abiko C Murata T Matsuzaki Y Itagaki T Sanjoh K Sakamoto M Hongo S Murayama S Hayasaka K Frequent Importation of enterovirus 71 from surrounding countries into the local community of Yamagata, Japan, between 1998 and 2003 J Clin Microbiol 2005 43 6171 6175 10.1128/JCM.43.12.6171-6175.2005 16333123 PMC1317214

[B24] Shimizu H Utama A Onnimala N Li C Li-Bi Z Yu-Jie M Pongsuwanna Y Miyamura T Molecular epidemiology of enterovirus 71 infection in the western Pacific region Pediatr Int 2004 46 231 235 10.1046/j.1442-200x.2004.01868.x 15056257

[B25] Yip CC Lau SK Zhou B Zhang MX Tsoi HW Chan KH Chen XC Woo PC Yuen KY Emenrgence of enterovirus 71 " double-recombinant" strains belonging to a novel genotype D originating from southern China: first evidence for combination of intratypic and intertypic recombination events in EV71 Arch Virol 2010 155 9 1413 1424 10.1007/s00705-010-0722-0 20549263 PMC7087135

[B26] Chan YF Sam IC AbuBakar S Phylogenetic designation of enterovirus 71 genotypes and subgenotypes using complete genome sequences Infect Genet Evol 2010 10 3 404 412 10.1016/j.meegid.2009.05.010 19465162

[B27] Tu PV Thao NT Perera D Huu TK Tien NT Thuong TC How OM Cardosa MJ McMinn PC Epidemiologic, virologic investigation of hand, foot, mouth disease, southern Vietnam, 2005 Emerg Infect Dis 2007 13 11 1733 1741 18217559 10.3201/eid1311.070632 PMC3375788

[B28] Perera D Yusof MA Podin Y Ooi MH Thao NT Wong KK Zaki A Chua KB Malik YA Tu PV Tien NT Puthavathana P McMinn PC Cardosa MJ Molecular phylogeny of modern coxsackievirus A16 Arch Virol 2007 152 1201 1208 10.1007/s00705-006-0934-5 17308978

[B29] ZHU Ru-nan QIAN Yuan DENG Jie XING Jiang-feng ZHAO Lin-qing WANG Fang LIAO Bin REN Xiao-xu LI Ying ZHANG Qi LI Jie Study on the association of hand, foot and mouth disease and enterovirus 71/CA16 among children in Beijing, 2007 Chin J Epidemiol 2007 28 10 1004 1008 18399150

[B30] Shih Shin-Ru Li Yi-Shuane Chiou Chiuan-Chian Suen Pin-Chau Lin Tzou-Yien Chang Luan-Yin Huang Yhu-Chering Tsao Kuo-Chien Ning Hsiao-Chen Wu Tzong-Zeng Chan Err-Cheng Expression fo caspid protein VP1 for use as antigen for the diagnosis of enterovirus 71 infection J Med Virol 2000 61 228 234 10.1002/(SICI)1096-9071(200006)61:2<228::AID-JMV9>3.0.CO;2-R 10797379

[B31] Chu PY Lin KH Hwang KP Chou LC Wang CF Shih SR Wang JR Shimada Y Ishiko H Molecular epidemiology of enterovirus 71 in Taiwan Arch Virol 2001 146 589 600 10.1007/s007050170164 11338392

[B32] AbuBakar S Chee HY AI-Kobaisi MF Xiaoshan J Chua KB Lam SK Identification of enterovirus 71 isolates from an outbreak of hand, foot and mouth disease (HFMD) with fatal cases of encephalomyelitis in Malaysia Virus Res 1999 61 1 1 9 10.1016/S0168-1702(99)00019-2 10426204

[B33] Perare D Podin Y Akin W Tan CS Cardosa MJ Incorrect identification of recent Asian strains of Coxsackievirus A16 as human enterovirus 71: improved primers for the specific detection of human enterovirus 71 by RT-PCR BMC Infect Dis 2004 4 11 10.1186/1471-2334-4-11 15122971 PMC415548

[B34] Rabenau HF Richter M Doerr HW Hand, foot and mouth disease: seroprevalence of Coxsackie A16 and Enterovirus 71 in Germany Med Microbiol Immunol 2010 199 1 45 51 10.1007/s00430-009-0133-6 19941005

[B35] Singh S Chow VT Phoon MC Chan KP Poh CL Direct detection of enterovirus 71 (EV71) in clinical specimens from a hand, foot, and mouth disease outbreak in Singapore by reverse transcription-PCR with universal enterovirus and EV71-specific primers J Clin Microlibol 2002 40 2823 2827 10.1128/JCM.40.8.2823-2827.2002 PMC120643 12149336

[B36] Kimura M A simple method for estimating evolutionary rates of base substitutions through comparative studies of nucleotide sequences J Mol Evol 1980 16 2 111 120 10.1007/BF01731581 7463489

