# Supplementary material for: Comparing Enterovirus 71 with Coxsackievirus A16 by analyzing nucleotide sequences and antigenicity of recombinant proteins of VP1s and VP4s
Source: BMC Microbiol. 2011 Nov 3;11:246. doi: 10.1186/1471-2180-11-246 (PMC3217892; doi:10.1186/1471-2180-11-246)
Supplement: Additional file 1 — The strains obtained from GenBank referred in this research. [file 1471-2180-11-246-S1.DOC]

### Additional file 1. The strains downloaded from GenBank referred in this research

| Isolate | Location (abbreviation) | Source | GenBank accession no.(Gene/s) |
| --- | --- | --- | --- |
| G-10 | South African(SAF) | GenBank | U05876(VP4, VP1) |
| UM16809 | Peninsular *Malaysia*(MAL) | GenBank | AM292535(VP4)  AM292483(VP1) |
| 2055 | Saudi Arabia (SA) | GenBank | AM292494(VP4)  AM292442(VP1) |
| S10432 | Sarawak, Malaysia (SAR) | GenBank | AM292507(VP4),  AM292455(VP1) |
| S70382 | Sarawak, Malaysia (SAR) | GenBank | AM292513(VP4),  AM292461(VP1) |
| CNS68762 | Sarawak, Malaysia (SAR) | GenBank | AM292503(VP4),  AM292451(VP1), |
| 5079 | Taiwan (TWN) | GenBank | AF177911(VP4,VP1), |
| 0033 | Perth, Western Australia (AUS) | GenBank | AM292487(VP4),  AM292435(VP1) |
| TS1-2000 | Thailand (THAI) | GenBank | AM292529(VP4),  AM292477(VP1) |
| S33071 | Sarawak, Malaysia (SAR) | GenBank | AM292510(VP4),  AM292458(VP1) |
| S114371 | Sarawak, Malaysia (SAR) | GenBank | AM292516(VP4),  AM292464(VP1) |
| 576T | South Vietnam | GenBank | AM292491(VP4),  AM292439(VP1) |
| UM15985 | Peninsular Malaysia (MAL) | GenBank | AM292534(VP4),  AM292482(VP1) |
| SB13044 | Sarawak, Malaysia (SAR) | GenBank | AM292527(VP4),  AM292475(VP1) |
| BrCr | USA | GenBank | U22521 |
| 06-KOR-00 | South Korea (KOR) | GenBank | DQ341355 |
| 2027-SIN-01 | Singapore (SIN) | GenBank | AY125993 |
| 8102-WA87 | USA | GenBank | AY123957 |
| BJ08-Z2025-5 | China (CHN) | GenBank | FJ606450 |
| Henan1-09-China | China (CHN) | GenBank | GU196833 |
| Zhejiang08 | China (CHN) | GenBank | Eu864507 |
| 3526-SIN-98 | Singapore (SIN) | GenBank | AY125984 |
| Anhui1-09-China | China (CHN) | GenBank | GQ994988 |
| Fuyang-0805 | China (CHN) | GenBank | FJ439769 |
| Henan2-09-China | China (CHN) | GenBank | GQ994992 |
| 1396-05-TW-EV71 | Taiwan (TW) | GenBank | DQ100443 |
| 4575-SIN-98 | Singapore (SIN) | GenBank | AY125986 |
| BJ08-Z020-1 | China (CHN) | GenBank | FJ606449 |
| Chongqing2-09-China | China (CHN) | GenBank | GQ994990 |
| China-GD7-2008 | China (CHN) | GenBank | FJ598111 |
| TW-2272-98 | Taiwan (TW) | GenBank | AF119795 |
| 2M-AUS-3-99 | Australia (AUS) | GenBank | AF376103 |
| 2120-SIN-01 | Singapore (SIN) | GenBank | AF376112 |
| 2222-IA-88 | USA | GenBank | AF009540 |
| 2609-AUS-74 | Australia (AUS) | GenBank | AF135886 |
| 2640-AUS-95 | Australia (AUS) | GenBank | AF135946 |
| 3799-SIN-98 | Singapore (SIN) | GenBank | DQ341354 |
| BJ08-Z020-01 | China (CHN) | GenBank | FJ606449 |
| F1-CHN-00 | China (CHN) | GenBank | Ab115490 |
| Fuyang22 | China (CHN) | GenBank | EU913466 |
| FY23 | China (CHN) | GenBank | EU812515 |
| KOR-EV71-3 | Korea (KOR) | GenBank | AY125968 |
| ZJ-CHN-6-03 | China (CHN) | GenBank | AY905619 |
| Tainan-4643-98 | Taiwan (TW) | GenBank | AF304458 |
| SHZH98 | China (CHN) | GenBank | AF302996 |
| SHZH03 | China (CHN) | GenBank | AY465356 |
| Shanghai036 | China (CHN) | GenBank | FJ713137 |
| 2235NY77 | USA | GenBank | AY123953 |
| 2007-07364 | Taiwan (TW) | GenBank | EU527983 |
| 2007-08747 | Taiwan (TW) | GenBank | EU527985 |
| 10076 | Netherlands | GenBank | AB575911 |
| 10857 | Netherlands | GenBank | AB575912 |
