# Supplementary material for: Comparing Enterovirus 71 with Coxsackievirus A16 by analyzing nucleotide sequences and antigenicity of recombinant proteins of VP1s and VP4s
Source: BMC Microbiol. 2011 Nov 3;11:246. doi: 10.1186/1471-2180-11-246 (PMC3217892; doi:10.1186/1471-2180-11-246)
Supplement: Additional file 2 — Virus strains cloned and sequenced in this research. [file 1471-2180-11-246-S2.DOC]

### Additional file 2. Virus strains cloned and sequenced in this research

| Isolate | Year | Location | Source | Type | GenBank accession no.(Gene/s) |
| --- | --- | --- | --- | --- | --- |
| S25 | 2007 | Beijing | This study | EV71 | JF317975(VP1), EU780699(VP4) |
| S47 | 2007 | Beijing | This study | EV71 | JF317976(VP1), EU780700(VP4) |
| F4211 | 2007 | Beijing | This study | EV71 | EU024958(VP1) |
| F4243 | 2007 | Beijing | This study | EV71 | EU019910(VP1), EU780703(VP4) |
| S65 | 2008 | Beijing | This study | EV71 | JF317977(VP1), EU780701(VP4) |
| S67 | 2008 | Beijing | This study | EV71 | JF317978(VP1), EU780702(VP4) |
| S97 | 2008 | Beijing | This study | EV71 | JF317979(VP1), EU862482(VP4) |
| S108 | 2008 | Beijing | This study | EV71 | JF317980(VP1) |
| S110b | 2008 | Beijing | This study | EV71 | JF317981(VP1), EU862483(VP4) |
| S110y | 2008 | Beijing | This study | EV71 | JF317982(VP1), EU862484(VP4) |
| S366 | 2009 | Beijing | This study | EV71 | JF317983(VP1), JF317987(VP4) |
| S374 | 2009 | Beijing | This study | EV71 | JF317984(VP1), JF317988(VP4) |
| S391 | 2009 | Beijing | This study | EV71 | JF317985(VP1) |
| S398 | 2009 | Beijing | This study | EV71 | JF317986(VP1) |
| S5 | 2007 | Beijing | This study | CA16 | JF317960(VP1) |
| S12 | 2007 | Beijing | This study | CA16 | JF317961(VP1) |
| S26 | 2007 | Beijing | This study | CA16 | JF317962(VP1) |
| S61 | 2007 | Beijing | This study | CA16 | JF317963(VP1) |
| S62 | 2008 | Beijing | This study | CA16 | JF317964(VP1) |
| S115 | 2008 | Beijing | This study | CA16 | JF317965(VP1) |
| S176 | 2008 | Beijing | This study | CA16 | JF317966(VP1) |
| S271 | 2008 | Beijing | This study | CA16 | JF317967(VP1) |
| S296 | 2008 | Beijing | This study | CA16 | JF317968(VP1) |
| S344 | 2009 | Beijing | This study | CA16 | JF317969(VP1) |
| S358 | 2009 | Beijing | This study | CA16 | JF317970(VP1) |
| S359 | 2009 | Beijing | This study | CA16 | JF317971(VP1) |
| S388 | 2009 | Beijing | This study | CA16 | JF317972(VP1) |
| S390 | 2009 | Beijing | This study | CA16 | JF317973(VP1) |
| S401 | 2009 | Beijing | This study | CA16 | JF317974(VP1) |
